# Route-resolved priority risks of disinfection by-products in indoor pools: the underestimated role of buccal/sublingual exposure for the public and competitive swimmers

**DOI:** 10.3389/fpubh.2026.1896577

**Published:** 2026-07-28

**Authors:** Mesut Genişoğlu, Mert Minaz, Bilgehan İlker Harman, Sait C. Sofuoglu

**Affiliations:** 1 Department of Environmental Engineering, Izmir Institute of Technology, Izmir, Türkiye; 2 Department of Aquaculture, Faculty of Fisheries, Recep Tayyip Erdoğan University, Rize, Türkiye; 3 Department of Environmental Engineering, Suleyman Demirel University, Isparta, Türkiye

**Keywords:** carcinogenic risk, disinfection byproducts, haloacetic acids, swimming pool, trihalomethanes

## Abstract

Disinfection of swimming pools is essential for preventing waterborne diseases; however, reactions between disinfectants and organic/inorganic precursors lead to the formation of disinfection byproducts (DBPs). Swimmers are exposed to these compounds through dermal contact, inhalation, accidental ingestion, and buccal/sublingual uptake. This study evaluated carcinogenic and chronic toxic risks associated with chloroform (CF), dichloroacetic acid (DCAA), and trichloroacetic acid (TCAA) using annual monitoring data from two indoor pools with contrasting operational characteristics (SP-A and SP-B). Results showed that inhalation of CF was the dominant carcinogenic pathway, consistently exceeding the *de minimis* level in all swimmer groups, particularly competitive swimmers. Oral pathways dominated HAA-related cancer risk, with TCAA producing the highest ingestion-route risks and exceeding the *de minimis* in competitive swimmers, reaching the low-priority risk range in SP-B. Dermal carcinogenic risks were considerable for CF, while DCAA and TCAA remained negligible owing to their low dermal permeability. Importantly, the buccal/sublingual route, often ignored in DBP assessments, yielded risks comparable to accidental ingestion, especially for children and high-intensity swimmers. Non-cancer risks associated with the estimated water-contact exposure routes were generally below the threshold. However, dermal exposure to CF in SP-B exceeded the threshold under some upper-bound and worst-case scenarios. Because non-cancer risks via the inhalation route could not be estimated due to the lack of an available reference concentration (RfC), non-cancer risks may be underestimated, as inhalation exposure was excluded from the assessment. In addition, both cancer and non-cancer risks may be further underestimated because only targeted DBPs were considered, whereas potential contributions from non-targeted and unidentified DBPs were not included in the risk assessment. Overall, DBP-related risks were higher in SP-B, emphasizing the influence of pool chemistry, bather load, and ventilation. These findings highlight the need for regular multi-route-specific risk assessment especially for competitive athletes and improved operational controls, particularly reducing TCAA precursors and enhancing ventilation for CF.

## Highlights

- Competitive swimmers exceed *de minimis* cancer risk level in high-DBP pools through ingestion and dermal route.
- Chloroform inhalation cancer risk exceeded *de minimis* level for all competitive swimmers.
- Non-cancer risks were generally low for quantified water-contact routes, except for dermal chloroform in high-DBP SP-B scenarios.
- Buccal/sublingual exposure may pose risks comparable to accidental ingestion.

## 1 Introduction

Swimming is widely recognized as a health-promoting activity due to its low-impact nature and full-body engagement, making it a globally popular sport (1). The frequency and length of activities in swimming pools tend to surpass those carried out at sea (2). Pools provide a controlled environment but also facilitate the accumulation of organic/inorganic compounds and pathogens, necessitating continuous disinfection to prevent disease outbreaks (3). While disinfectants used in swimming pools neutralize pathogens, disinfection byproducts (DBPs) are formed through reactions with natural organic matters (NOM) and anthropogenic organic matters (e.g., human excretion, hair, pharmaceuticals, and personal care products). Commonly used disinfection methods include chlorine-based disinfectants, bromine, ozone, and UV (4).

Since the discovery of trihalomethanes (THMs) in drinking water in the 1970s and in swimming pools in the 1980s (5, 6), numerous studies have examined the occurrence of DBPs. Beyond THMs, numerous DBP classes including HAAs, haloaldehydes, halonitriles, nitrosamines, and others have been detected in swimming pools (4, 7, 8). Among these, THMs (e.g., chloroform, bromodichloromethane) and HAAs are the most prevalent and are widely used as DBP indicators in chlorinated waters (9, 10). Recent prioritization studies have emphasized that commonly occurring and toxicologically relevant DBPs in indoor swimming pools should be identified using tier-based screening frameworks. This approach supports the use of priority compound selection in pool-specific risk assessments, particularly when the full DBP mixture cannot be evaluated quantitatively (11). To date, more than 700 DBPs, many with cytotoxic, genotoxic, or carcinogenic potential, have been identified in disinfected waters (8, 12).

DBP species and concentrations in swimming pools depend on factors such as disinfectant type and dose, source water quality, swimmer load, age, sex, and hygiene (13, 14). Operational conditions such as residual disinfectant, reaction time, temperature, and pH also influence DBP formation (3). The amount of organic loads is determined by several factors such as temperature and swimmer hygiene (15). Ammonia, urea, creatinine, citric acid, uric acid, gluconic acid, various amino acids, and sodium chloride, which are components of urine and sweat as human excretion, are quite common in swimming pools (16). A 30-min swim releases approximately 250 mg non-purgable organic carbon (NPOC), 77.3 mg total nitrogen, 37.1 mg urea, and 10.1 mg ammonium per bather, with sebum accounting for a substantial fraction of NPOC (13). Tsamba et al. (17) emphasize that the biochemical diversity of these inputs, including sweat, urine, sebum, and personal care products, complicates efforts to characterize precursors in pools and establish mechanistic pathways for DBP formation. Indeed, one of the major difficulties in understanding DBPs formation in pools is the complex composition of the precursors released by bathers.

Although swimming is widely recommended as a health-promoting physical activity (18–20), pool water has been reported to exhibit greater cytotoxic and mutagenic potential compared to municipal drinking water (21). While the most commonly reported adverse effects among swimmers are short-term and irritative, such as cough (84%), eye irritation (78%) and itching (34%) (22), long-term exposure to THMs through dermal absorption, inhalation, and/or ingestion during showering and swimming has been associated with an elevated risk of bladder cancer (23). Children represent a particularly vulnerable population due to several factors: lower body weight, higher accidental ingestion rate and higher inhalation rate relative to body weight, all of which result in higher exposure doses and consequently elevated health risks (24). Thus, the DBP-associated health risks extend beyond exposure through pool water. Due to the high volatility of several DBP compounds, inhalation constitutes a significant exposure pathway and is therefore a critical consideration for public health protection. Therefore, comprehensive human health risk assessments should account for DBP exposure across both aqueous and airborne phases.

Buccal and sublingual exposure pathways have rarely been evaluated separately in swimmer DBP risk assessments, although the vascularized structure of the oral mucosa may allow direct uptake during repeated mouth–water contact (25). In previous swimming-pool risk assessments, buccal and aural exposure pathways were reported to contribute substantially to total DBP-related risk, accounting for approximately 17.9–38.9% of the estimated total risk. Therefore, exclusion of non-ingestive oral exposure may result in underestimation of aggregate swimmer exposure, particularly in high-contact swimming scenarios (26). As a result, these routes are underrepresented in multi-route DBP assessments, leading to underestimated aggregate risk. Although DBP occurrence in swimming pools has been investigated in Türkiye, country-specific information on route-resolved DBP exposure and health-risk characterization remains limited, particularly for combined oral, dermal, inhalation, and buccal/sublingual pathways. It is essential to characterize swimmer risks under country-specific conditions. With the nationwide “No One Left Who Does not Know How to Swim” initiative training more than 8 million children, indoor pool use has expanded rapidly, increasing the importance of evaluating DBP-related health risks. Continuous disinfection combined with high bather loads can generate carcinogenic, toxic, and irritant DBPs, posing heightened risks to sensitive groups (27).

This study provides a route-resolved DBP risk assessment by incorporating buccal and sublingual pathways, which are often excluded from swimmer exposure assessments, alongside accidental ingestion, dermal contact, and inhalation. To our knowledge, this is the first study in Türkiye to evaluate DBP-related health risks in indoor pools using a multi-route framework that includes buccal/sublingual exposure. Building on year-round THM and HAA monitoring data from two pools fed by distinct source waters (28), the study evaluates carcinogenic and chronic-toxic risks across swimmer types, age groups, and exposure scenarios, addressing both national and international gaps in multi-route DBP risk characterization.

## 2 Materials and methods

The DBP concentrations used for the risk assessment were measured over a year in two different indoor swimming pools, as reported in our previous study (28), with mean water concentrations of CF, DCAA, and TCAA of 19.6, 23.9, and 25.7 μg/L in SP-A and 68.9, 56.1, and 325 μg/L in SP-B, respectively (see Supplementary Table S1). Before the sampling campaign, both pools were emptied, cleaned, and refilled with fresh source water. The source water for swimming pool B (SP-B) was groundwater, whereas chlorinated municipal drinking water was used to fill swimming pool A (SP-A). The dimensions of SP-A were 50 m × 25 m × 2.33 m(H), while SP-B were 25 m × 16 m × 2.00 m(H). Settled particles in both pools and their buffer tanks were removed using a robotic cleaner. Pool water filtration was achieved using sand filters: SP-A was equipped with six sand filters, each with a filtration cross-sectional area of 3.14 m^2^ and operating at a working pressure of 35.5 PSI. SP-B utilized eight sand filters, each with a filtration cross-sectional area of 0.64 m^2^ and a working pressure of 28.4 PSI.

The pools remained closed on Mondays for cleaning and maintenance, operating from Tuesday to Sunday. The average weekly swimmer load (Tuesday to Sunday) was 1,350 for SP-A and 950 for SP-B, with total swimmer counts during the sampling campaign recorded at 64,704 and 45,423, respectively. SP-A is open for university students and academics, while SP-B serves as a public facility, attracting a high number of child swimmers, particularly during the summer. Pool water samples were collected on Sunday evening to represent the weekly load before the cleaning day. A total of 48 weekly sampling events were conducted for each pool during the annual monitoring period. Summary statistics for the measured DBP concentrations, including mean, standard deviation, median, and percentile values, are provided in Supplementary Table S1. Water samples were collected 20 cm below from the water surface and 50 cm away from the edges. The average TOC, pH, and free chlorine levels in SP-A were determined to be 1.52 mg/L, 7.68, and 0.39 mg/L, respectively, while those in SP-B were 2.51 mg/L, 7.49, and 0.64 mg/L. The details of physico-chemical compositions of sample pools were given in Genisoglu et al. (28). Analysis of THMs and HAAs were performed after liquid–liquid extraction using a gas chromatography with an electron capture detector (GC-ECD, Agilent 6,890).

### 2.1 Risk assessment

The methodology recommended by the USEPA was used for health risk assessment (29). Lifetime cancer risks (CR) associated with dermal contact, inhalation, and ingestion exposure routes were estimated using Equations 1–3, respectively. The USEPA’s *de minimis* risk threshold of 10^−6^, corresponding to a risk of no more than one in a million, is commonly adopted as the demarcation criterion for defining a safe zone. Because no swimming-pool-specific regulatory classification is available for route-resolved DBP cancer risks, CR values were interpreted using the de minimis benchmark of 10^−6^ together with the prioritization ranges proposed by Legay et al. (30). These categories were used only as screening-level risk-communication and prioritization tools, not as definitive regulatory safety thresholds. Accordingly, CR ≤ 10^−6^ was described as below the de minimis benchmark, 10^−6^ < CR < 10^−5^ as an acceptable risk, 10^−5^ < CR < 10^−4^ as a low-priority risk, and CR ≥ 10^−4^ as a higher-priority risk range requiring closer risk-management attention.

Gas phase concentrations of chloroform were estimated using a regression model (Equation 4, R^2^ = 0.815) derived by Dyck et al. (31). The model was evaluated against literature-reported air–water concentration measurements and showed good agreement for chloroform, with normalized mean bias (NMB) and mean fractional bias (MFB) values ranging from 1.7 to 17%, indicating limited systematic deviation between predicted and observed concentrations. However, HAAs were not considered due to their non-volatility. Indoor air was assumed to be homogeneous, and chloroform concentrations were considered equal at all points.

Hazard Quotient (HQ) is used to evaluate non-cancer chronic hazard potential (Non-Cancer Risk; non-CR) based on estimated exposures. HQ is defined as the ratio of the potential dose to the Reference Dose (RfD, mg/kg-day), the dose below which no adverse health effects are expected (Equations 5, 6). In this study, chronic hazard levels are interpreted as follows: HQ ≤ 0.1 indicates no anticipated adverse effects; 0.1 < HQ ≤ 1 suggests a potentially meaningful hazard that warrants further examination; HQ > 1 indicates a clear hazard potential, while HQ > 2 reflects considerable concern (32). Non-cancer risk associated with inhalation exposure was not estimated because compound-specific reference concentrations or inhalation reference doses were not available for the target DBPs in the Integrated Risk Information System or the Risk Assessment Information System. Therefore, non-cancer risk estimates were limited to ingestion, dermal contact, and buccal/sublingual exposure routes (33). Values of the constants used in risk estimation for the three DBPs are listed in Supplementary Table S2 (33–36). In addition, aggregate risks were calculated as the sum of risks derived from different exposure route for each DBP.

$$CR_{\mathrm{ing}}=\mathrm{SF}\times (\frac{\text{IngR}\times C_{w}\times \mathrm{ED}\times \mathrm{EF}}{\mathrm{AT}\times \mathrm{BW}})$$ (1)

$$CR_{\text{dermal}}=\mathrm{SF}\times (\frac{\mathrm{SA}\times K_{p}\times C_{w}\times \mathrm{ED}\times \mathrm{EF}}{\mathrm{AT}\times \mathrm{BW}})$$ (2)

$$CR_{\mathrm{inh}}=\mathrm{SF}\times (\frac{C_{\mathrm{air}}\times \mathrm{IR}\times \mathrm{ED}\times \mathrm{EF}}{\mathrm{AT}\times \mathrm{BW}})$$ (3)

$$C_{\mathrm{air}*}=4.229xC_{w*}-0.039$$ (4)

$$HQ_{\mathrm{ing}}=\frac{\text{IngR}\times C_{w}\times \mathrm{ED}\times \mathrm{EF}}{\mathrm{AT}\times \mathrm{BW}}/\mathrm{RfD}$$ (5)

$$HQ_{\text{Dermal}}=\frac{\mathrm{SA}\times K_{p}\times C_{w}\times \mathrm{ED}\times \mathrm{EF}}{\mathrm{AT}\times \mathrm{BW}}/\mathrm{RfD}$$ (6)

where, *CR*: carcinogenic risk, SF : slope factor (mg/kg-d); $C_{w}$ : concentration in water (mg/L); $C_{w*}$ : μg/L; *IngR*: ingestion rate of water (L/h); *IngR* replaced by *WI×AR_Buccal/Sublingual_* (water intake for buccal and sublingual pathway, L/h × absorption factor for buccal and sublingual pathway) for CR_Buccal/Sublingual_; *EF*: exposure frequency (h/year); *ED*: exposure duration (year); *SA*: skin surface area (m^2^); *Kp*: specific permeability coefficient (cm/h); $C_{\mathrm{air}}$ : concentration in air (mg/m^3^); $C_{\mathrm{air}*}$ : μg/m^3^; *IR*: inhalation rate (m^3^/h); *BW*: body weight (kg); *AT*: average lifetime (day), *HQ*: Hazard index, *RfD*: reference dose (mg/kg-d), AT assumed to be equal to ED × 365 days while estimating HQ. In Equation 4, Cw represents the chloroform concentration in pool water expressed in μg/L, and Cair represents the estimated chloroform concentration in indoor pool air expressed in μg/m^3^.

For the buccal/sublingual pathway, IngR was replaced by WI × ARBuccal/Sublingual, where WI represents the non-ingested water-contact parameter for the buccal/sublingual route and ARBuccal/Sublingual represents the mucosal absorption factor. WI values were 1.25 L/h for competitive adults, 2.50 L/h for competitive children, 2.50 L/h for non-competitive adults, and 5.00 L/h for non-competitive children (Supplementary Table S3). Accidental ingestion refers to swallowed pool water, whereas buccal/sublingual exposure represents water retained in contact with the oral mucosa and then expelled; therefore, only the absorbed fraction, WI × ARBuccal/Sublingual, was considered for this pathway.

To ensure unit consistency, all water-phase DBP concentrations were converted from μg/L to mg/L using a factor of 10^−3^, and modeled indoor-air CF concentrations were converted from μg/m^3^ to mg/m^3^ using a factor of 10^−3^. For dermal calculations, skin surface area was converted from m^2^ to cm^2^ using a factor of 10^4^ to match the permeability coefficient units (cm/h), and a conversion factor of 10^−3^ L/cm^3^ was applied to convert the water volume term generated by Kp × SA × time into liters. Thus, route-specific average daily doses were expressed as mg/kg-day, and the resulting cancer risks and hazard quotients were dimensionless. Route-specific toxicity values were used where available. For CF inhalation cancer risk, an inhalation slope factor derived from the inhalation unit risk was used; oral slope factors were not used for CF inhalation. Inhalation cancer risks for DCAA and TCAA were not estimated because compound-specific inhalation unit risks or inhalation slope factors were not available. Oral slope factors were used for accidental ingestion and buccal/sublingual exposure, while buccal/sublingual absorption was treated as a screening-level oral-mucosal uptake scenario due to the absence of DBP-specific buccal/sublingual toxicity factors.

### 2.2 Exposure assessment

Three routes of exposure, dermal contact, inhalation, and ingestion (accidentally ingested water during swimming) were investigated in this study. Three age groups (adults, children aged 7–10 years “C 7–10” and 11–14 years “C 11–14”) in two swimmer groups (competitive and non-competitive) were considered in central-tendancy and worst-case scenarios. The scenario-specific exposure-related variables used for central-tendency and worst-case calculations are listed in the revised Supplementary Table S3 (35–40). The WI values used for buccal/sublingual exposure differ from accidental ingestion rates because WI represents non-ingested oral water contact rather than swallowed water volume. The most up-to-date exposure factors appropriate to the available age groups were used. Average values of the independent input variables in Supplementary Table S3 were used for the central-tendency scenario, whereas upper-bound values (95th percentile when available) were used for the worst-case scenario. Body surface areas of swimmers were calculated using Du Bois empirical model [see Supplementary material (Equation S1)] (40, 41). The average height and weight of Turkish people (39) were used in the calculations.

### 2.3 Uncertainities

Uncertainty is an inherent aspect of all exposure and risk assessments, and several limitations should be acknowledged in the present evaluation (42). Activity-budget parameters were taken from internationally reported swimmer datasets rather than Turkish population-specific behavioral data, which aligns exposure estimates with global reference patterns but reduces their representativeness for local swimmers. Temporal variability in DBP concentrations, driven by bather load, chlorination demand, water replacement, and ventilation conditions, may not be fully captured by the sampling design. Samples were collected weekly on Sunday evenings to represent the accumulated weekly load before cleaning; therefore, the dataset reflects a conservative weekly pre-cleaning condition rather than full day-to-day or within-day variability. Seasonal and inter-week variability were partially represented by the annual monitoring period, but short-term temporal fluctuations could not be fully evaluated. Additional uncertainty arises from physiological input parameters (body weight, skin surface area, inhalation rate), which rely on default reference values and may not reflect the heterogeneity of children, adolescents, and competitive swimmers. Dermal permeability coefficients, particularly for HAAs, are based on limited experimental data and may not fully represent age- or site-specific skin characteristics. For the buccal/sublingual route, the absorption factor (AR = 0.01) was adopted from the U. S. EPA SWIMODEL framework, which includes buccal/sublingual and aural exposure routes as part of its full swimmer-exposure calculation structure. Because this AR value is based on nitroglycerin rather than DBP-specific oral-mucosal permeability data, the buccal/sublingual risk estimates should be interpreted as screening-level, order-of-magnitude approximations rather than precise route-specific predictions (35). Because the indoor air CF concentrations used for risk estimation were estimated from water concentrations using an empirical regression model, some degree of bias cannot be ruled out due to moderate regression coefficient and potentially arising from differences in pool operational parameters, building characteristics, mechanical ventilation systems, treatment processes, and bather load between model calibration pool and ours that could have influenced the modeled indoor air concentrations. The applied model was developed and evaluated by Dyck et al. (31), who reported good agreement with measured air–water concentration data and identified key drivers of variability such as air exchange rate, water concentration, swimming duration, inter-media transport coefficient, and Henry’s law constant in their sensitivity analysis. These parameters represent important sources of uncertainty in indoor air exposure estimation and should be considered in future site-specific sensitivity analyses. Furthermore, because DCAA and TCAA exhibit very low volatility, their pool-air concentrations were not modeled, and aerosol-driven inhalation exposure during splashing may be underestimated; inhalation risks for these HAAs could not be quantified in any case due to the absence of inhalation unit-risk values or slope factors. Finally, cumulative risk estimation is constrained by differences in mode of action across DBP classes, limiting risk addition to chemical groups with compatible toxicological mechanisms. Because the quantitative assessment was restricted to CF, DCAA, and TCAA, the results do not capture potential risks from other THMs, additional HAAs, brominated DBPs, nitrogenous DBPs, chloramines, trichloramine, or other volatile and irritant compounds relevant to swimming-pool air. In particular, the absence of chloramine/trichloramine measurements limits the interpretation of respiratory irritation, occupational exposure, and pool-air-related public-health risks for swimmers, coaches, and pool staff. Therefore, the results should be interpreted as priority screening estimates for selected DBPs rather than as a complete assessment of total DBP mixture risk. Collectively, these factors indicate that the reported non-CR levels should be interpreted as bounded, approximate estimates, suitable for comparative assessments and risk-management prioritization rather than precise predictors of individual health outcomes.

## 3 Results and discussion

Descriptive statistics for the measured water concentrations and modeled indoor air concentrations in both pools are presented in Supplementary Table S1. Only DBPs detected in more than 50% of samples and for which suitable toxicity values were available were included in the quantitative risk assessment (Supplementary Table S4). Supplementary Table S4 also summarizes the detection frequencies of the measured THMs and HAAs in SP-A and SP-B and provides the inclusion/exclusion decision for each compound. Accordingly, the present analysis should be interpreted as a priority screening assessment focused on CF, DCAA, and TCAA, all of which were detected in 100% of samples and had available risk factors for the evaluated routes. The method detection limit was 0.30 μg/L for both THMs and HAAs, and concentrations below the MDL were treated as non-detects for the purpose of detection-frequency screening. This compound-selection strategy was used to enable route-resolved risk estimation, but it does not represent the full DBP mixture present in indoor pool environments. Carcinogenic risks calculated for the three routes of exposure, i.e., ingestion, dermal contact, and inhalation, are presented in the following sections.

### 3.1 Ingestion route carcinogenic risk

**Figure 1:**
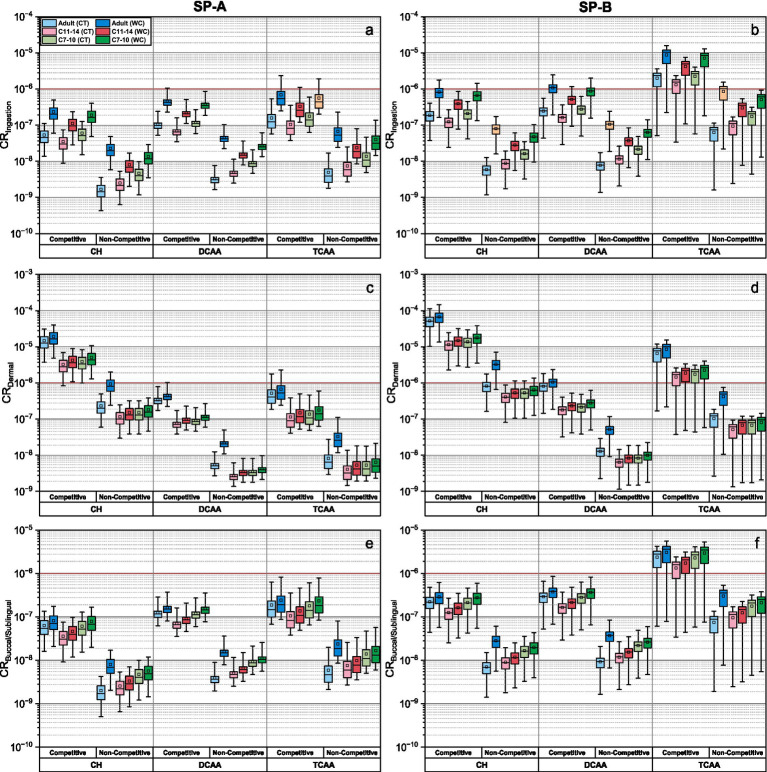
Estimated CRs for swimmers through ingestion **(a,b)**, dermal exposure **(c,d)**, and buccal/sublingual routes **(e,f)**. Panels on the left show CRs for SP-A, and panels on the right show CRs for SP-B.

CR levels associated with ingestion exposure to CF, DCAA, and TCAA are shown in Figures 1a,b. The estimated average ingestion route CR levels of CF, DCAA, and TCAA in SP-A for central tendency scenario were ranged from 1.68 × 10^−9^ to 1.76 × 10^−7^, while those in SP-B were ranged from 5.90 × 10^−9^ to 2.23 × 10^−6^. Those levels for worst-case scenario were ranged between 7.99 × 10^−9^ – 6.89 × 10^−7^ and 2.81 × 10^−8^ – 8.71 × 10^−6^, respectively. CR in SP-B were relatively higher than SP A for all scenarios. The estimated worst case ingestion route CR for DCAA in competitive adults and for TCAA in all competitive swimmers were exceeded the *de minimis* risk level. However, these risks remained within the acceptable risk range. In contrast, the ingestion route CR levels for all other swimmer groups were below the *de minimis* even under the worst-case exposure scenarios.

Ingestion-route CRs differed by pool, compound, swimmer category, age group, and exposure scenario. Overall, SP-B produced higher ingestion-route CRs than SP-A, consistent with its higher measured DBP concentrations. Among the evaluated DBPs, TCAA was the main contributor to ingestion-related cancer risk, followed by DCAA and CF. Exceedances of the de minimis benchmark were mainly observed under worst-case assumptions and were concentrated in competitive swimmers, particularly for TCAA in SP-B. In contrast, most non-competitive swimmer scenarios remained below the de minimis benchmark.

A consistent and substantial swimmer-type effect was observed. Regardless of chemical or pool, competitive swimmers exhibited markedly higher CR levels than non-competitive swimmers due to higher ingestion volumes and longer effective exposure durations. Cross-chemical comparisons further indicated that TCAA produced the highest ingestion-route cancer risks overall, with DCAA consistently higher than CF, and TCAA exceeding both, particularly in SP-B where TCAA reached levels nearly an order of magnitude above CF. Collectively, these findings demonstrate that ingestion-related cancer risk is strongly dependent on pool conditions (SP-B > SP-A) and swimmer behavior (competitive > non-competitive), with TCAA in SP-B competitive swimmers representing the only scenario reaching the low-priority risk range. If the management objective is to maintain all ingestion-route CR values at or below 10^−6^, targeted DBP control, particularly reduction of TCAA precursors and optimization of chlorination conditions in SP-B, emerges as a critical intervention. Median CR values were predominantly within the safe zone, with the exception of competitive swimmers exposed to TCAA; even at the upper bound level, most scenarios still fell within acceptable or safe risk levels, indicating limited ingestion-related concern outside TCAA-driven competitive cases.

The higher ingestion-route cancer risk associated with TCAA is primarily attributed to its larger oral cancer slope factor and its higher concentrations in pool water, reflecting its chemical stability and persistence. Differences between SP-A and SP-B may furthermore be influenced by the efficiency of filtration and circulation systems, the quality and origin of filling water, and variations in swimmer load and swimmer profile. Such variation aligns with the well-established differences in natural organic matter (NOM) composition across water sources: groundwater typically has lower humic and fulvic content and therefore a lower DBP formation potential, whereas surface waters contain higher levels of high-molecular-weight humic substances that substantially enhance DBP formation (43). NOM residues and DBPs carried from drinking water distribution systems further contribute both precursors and DBP loads to swimming pools, while untreated source-water NOM remains a key driver of DBP formation (7). Swimmer profile also plays a critical role, as children introduce substantial TOC through sweat, body oils, and urine, amplified by high physical activity and insufficient pre-swim hygiene practices (44).

Although previous studies report higher accidental ingestion rates for non-competitive swimmers compared with competitive swimmers (38), the present results indicate that ingestion-route cancer risks were substantially higher in competitive swimmers due to their longer exposure duration. Across chemicals, TCAA yielded the highest ingestion-route cancer risk, followed by DCAA and CF, in relation to their relative toxicity and concentration profiles in pool water. In SP-A, cancer risks from all DBPs and all exposure routes were estimated to be in the no-risk category, below the *de minimis* level. In contrast, exceedances of the *de minimis* threshold were more frequent in SP-B, particularly for DCAA and TCAA. While the estimated ingestion route CRs for CF and DCAA remained within the acceptable risk range, TCAA exhibited the highest risk estimates. For TCAA, ingestion route CR values exceeded the *de minimis* threshold in all competitive swimmer groups under both the central tendency and worst-case scenarios, reaching 1.99 × 10^−6^ and 8.71 × 10^−6^ in adults, 2.23 × 10^−6^ and 7.08 × 10^−6^ in the 7–10 age group, and 1.31 × 10^−6^ and 4.16 × 10^−6^ in the 11–14 age group, respectively. Nevertheless, all estimated risks remained within the acceptable risk range. All remaining non-competitive swimmer groups remained below the *de minimis* threshold. These findings align with previous literature reporting ingestion-route cancer risks for CF in the range of 10^−7^–10^−9^ and DCAA risks near the *de minimis* level (1, 44, 45). The lifetime average daily dose of CF through ingestion (~10^−6^ mg/kg-day) reported by Abbasnia et al. (46) also matches the exposure magnitudes calculated in this study when combined with its oral slope factor. The exceedances associated with TCAA in competitive swimmers, and to a no excedence in non-competitive adults even under worst-case conditions, indicate that pools with insufficient DBP control may accumulate TCAA to levels capable of producing ingestion-route cancer risks above acceptable limits. This interpretation is consistent with prior evidence identifying water concentration, exercise duration, and body weight as dominant determinants of ingestion-route exposure (31).

### 3.2 Dermal contact route carcinogenic risk

Figures 1c, d shows the estimated dermal route CR, revealing patterns of pool difference that closely parallel the ingestion-route framework while highlighting the influence of pool-specific parameters on risk magnitude. Across all scenarios, dermal cancer risks were consistently higher in SP-B competitive swimmers than in any swimmer group in SP-A. This trend was further reinforced by the prolonged exposure durations characteristic of competitive training sessions, as expected given the higher concentrations in SP-B, collectively underscoring the strong sensitivity of CR outcomes to exposure parameters. In the Adult–Competitive worst-case, the median CR under SP-B reached 6.60 × 10^−5^ and the upper-bound level rose to 1.08 × 10^−4^, the former falling within the low-priority risk zone and the latter exceeding the cutoff for unacceptable risk. The corresponding CR in SP-A remained entirely within the low-priority risk range, indicating that dermal exposure under both conditions warrants attention despite not uniformly reaching high-priority management thresholds. In children, median CR levels were lower; nevertheless, Competitive–SP-B scenarios for both the 7–10 and 11–14 age groups estimated within the low-priority risk zone while those were acceptable in SP-A. Although CF is traditionally characterized by a dominant inhalation pathway, the strong correspondence between dermal and ingestion trends indicates that dermal uptake meaningfully contributes to cancer risk under realistic recreational exposures.

DCAA, by contrast, produced low absolute dermal CR values that fall primarily within no-risk or acceptable-risk categories according to the previous classification. However, dermal CR estimates displayed a consistent upward shift under SP-B, with mean and upper-bound levels increasing by approximately two- to three-fold relative to SP-A. While DCAA does not reach levels of regulatory concern in absolute magnitude, its pronounced responsiveness to exposure-model configuration highlights the susceptibility of ionizable, low-dose HAAs to changes in assumptions governing water, skin exchange, contact duration, and scenario structure, an effect also observed for DCAA in the ingestion pathway. This methodological sensitivity does not elevate DCAA into higher-risk categories but signals the need for caution when comparing dermal CR across studies or integrating multi-pathway cancer risk.

Despite its low absolute dermal CR, the ~11-fold amplification of TCAA in SP-B reflects its marked responsiveness to pool-specific DBP loading, making it a chemically sensitive indicator in high-DBP pools. Competitive scenarios produced the strongest responses, particularly in younger swimmers, whose higher surface-area-to-mass ratios intensify the effect of water chemistry and contact duration. This exaggerated scenario-dependence parallels TCAA’s ingestion-route behavior and underscores its status as a DBP for which risk assessments are especially model-dependent; transparency in exposure assumptions is therefore essential for reproducibility and cross-study comparability.

Across both ingestion and dermal pathways, competitive swimmers generally showed higher exposure potentials due to longer time spent in the pool; however, the magnitude of competitive–non-competitive differences diverged sharply between pathways (Figures 1a–d). Oral-route cancer risks were strongly influenced by the scenario-specific assumptions for session duration, ingestion rate, and buccal/sublingual water contact, as summarized in the revised Supplementary Table S3. Therefore, competitive and non-competitive swimmer groups were compared separately under central-tendency and worst-case assumptions rather than being interpreted as a uniformly higher-risk group across all oral-exposure scenarios. Dermal uptake, in contrast, scales nearly linearly with immersion time, leading to substantially larger scenario-dependent differences for pool water contact than for ingestion.

Consistent with earlier evidence reporting dermal chloroform risks between 10^−9^–10^−7^ and, in some cases, 10^−5^–10^−7^ (1, 44, 46, 47), our results similarly indicate elevated dermal CR under competitive swimming conditions. The pronounced SP-B/SP-A amplification further widened the competitive–non-competitive gap, with CF showing the largest divergence (∆Mean = 6.4 × 10^−5^), followed by smaller yet meaningful differences for DCAA (9.99 × 10^−7^) and TCAA (8.06 × 10^−6^). Notably, previous research has also shown demographic sensitivity in dermal HAA exposure, DCAA-based dermal cancer risk in indoor pools was highest in women, followed by men and children (48), supporting the view that physiological and behavioral factors can modulate dermal uptake. This outcome aligns with prior findings indicating that dermal exposure can dominate total risk, with one study attributing 94.2% of aggregate CR to dermal uptake (49).

### 3.3 Buccal/sublingual route carcinogenic risk

CR estimates through buccal/sublingual exposure route ranked as TCAA > DCAA > CF, reflecting compound-specific potency and their differing capacities to contribute to cumulative cancer risk (Figures 1e,f). TCAA-related carcinogenic risks in competitive adults rose from 1.87 × 10^−7^ (central-tendancy) to 2.43 × 10^−7^ (worst-case) in SP-A, and from 2.37 × 10^−6^ (central-tendancy) to 3.07 × 10^−6^ (worst-case) in SP-B. Comparable scenario-dependent increases were observed for DCAA and CF, demonstrating that water-saturated conditions and intensified swimmer–water interaction markedly elevate carcinogenic risk through the buccal/sublingual route.

The largest contrasts between competitive and non-competitive swimmers occurred for TCAA under worst-case scenarios, where carcinogenic risks in competitive adults approached an order of magnitude above those in non-competitive individuals. These differences underscore the contribution of the frequency of mouth–water interaction to overall cancer risk.

CR distributions were positively skewed, with upper-bound levels typically 1.6–3.2 times higher than the means. Skewness was most pronounced for TCAA in competitive swimmers under worst-case scenario (upper-bound level up to 5.09 × 10^−6^), revealing the presence of high-risk subgroups. Systematic differences between estimation frameworks were also observed, with SP-B producing 2.5–15-fold higher CRs than SP-A, identifying SP-B as a conservative upper-bound carcinogenic risk estimator.

To evaluate the uncertainty associated with the buccal/sublingual absorption factor (AR), a Monte Carlo–based uncertainty analysis was performed using Crystal Ball software (Oracle). Due to the lack of DBP-specific oral mucosal absorption data, AR was assumed to follow a uniform distribution between 0.001 and 0.1, representing one order of magnitude below and above the default SWIMODEL value (AR = 0.01). The analysis was conducted using 200 simulations with 1,000 trials per simulation, and the resulting distributions were used to quantify variability in both buccal/sublingual and aggregate cancer risk estimates for CF. The uncertainty was expressed in terms of coefficient of variation (CV) for the forecasted cancer risk. The buccal/sublingual CF cancer risk exhibited a CV of 0.57 ± 0.01 (range: 0.54–0.60), whereas the aggregate CF cancer risk showed a CV of 1.16 × 10^−2^ ± 1.80 × 10^−4^ (range: 1.12 × 10^−2^–1.20 × 10^−2^), indicating a moderate effect of AR uncertainty on route-specific risk and a negligible effect on total aggregate risk. As AR was the only varying parameter in the bootstrap analysis for CF, similar parameter-driven uncertainties may also exist for other stressors with comparable input dependencies.

Although buccal/sublingual uptake presents a meaningful carcinogenic risk, swimmer exposure studies in the literature predominantly prioritize dermal absorption, accidental ingestion, and inhalation, with buccal/sublingual carcinogenic risk largely overlooked in swimmers health risk assessments. The present findings demonstrate that buccal/sublingual CRs can approach, and under worst-case scenarios exceed, those typically attributed to accidental ingestion, indicating that the exclusion of this pathway leads to systematic underestimation of aggregate carcinogenic risk, particularly in high-intensity swimming or during prolonged face–water contact. Moreover, even though buccal/sublingual intake rates are lower in competitive swimmers, the substantially longer and more intensive training durations characteristic of competitive swimmers result in higher oral mucosal exposure, ultimately yielding elevated buccal/sublingual carcinogenic risk levels relative to their non-competitive counterparts.

### 3.4 Inhalation route carcinogenic risk

**Figure 2:**
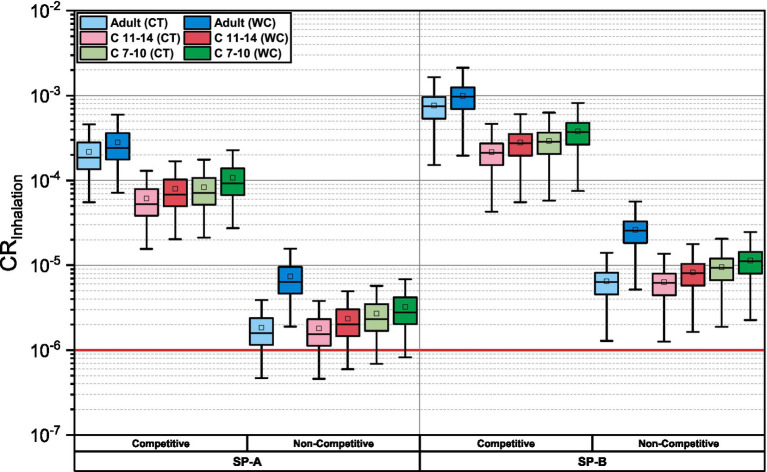
Estimated CRs of CF for swimmers through inhalation route.

Carcinogenic risks estimated for inhalation exposure are presented in Figure 2. Competitive adult swimmers exhibited the highest inhalation route CRs, with values ranging from 2.17 × 10^−4^ to 2.81 × 10^−4^ in SP-A and 7.62 × 10^−4^ to 9.88 × 10^−4^ in SP-B, clearly exceeding the commonly referenced 10^−4^ management threshold. Competitive C 11–14 swimmers showed intermediate risk levels (6.12 × 10^−5^–7.94 × 10^−5^ in SP-A and 2.16 × 10^−4^–2.79 × 10^−4^ in SP-B), followed closely by competitive C 7–10 swimmers, whose risks reached 8.28 × 10^−5^–1.07 × 10^−4^ in SP-A and 2.92 × 10^−4^–3.78 × 10^−4^ in SP-B. In all competitive subgroups, SP-B risks were approximately 3.5-fold higher than SP-A, consistent across central-tendancy and worst-case scenarios. For non-competitive swimmers, risks remained markedly lower and mostly within the 10^−6^–10^−5^ range in SP-A, and 10^−5^–10^−4^ in SP-B. As expected, adult swimmers demonstrated higher inhalation risks than younger age groups because of their higher inhalation rates and exposure durations.

Given its higher volatility (41), CF continues to dominate inhalation risk across all swimmer groups. Previous studies have similarly reported elevated blood chloroform concentrations in swimmers without breathing equipment compared to control groups, reflecting the strong likelihood of inhalation exposure to volatile DBPs in pool environments (10). In swimming-pool environments, THM uptake via inhalation has been estimated to contribute a higher cancer risk than ingestion or dermal exposure, particularly for volatile compounds such as chloroform (43). Epidemiological evidence from Spain has also highlighted an increased risk of chronic lymphocytic leukemia associated with THM exposure among swimmers (50).

Average inhalation cancer risks of CF remained >*de minimis* level for all swimmer groups, and frequently much higher for competitive swimmers, particularly adults, reinforcing that inhalation represents the dominant route of DBP exposure in indoor swimming pools where volatile DBPs and aerosolized droplets accumulate above the water surface (51–53). Competitive swimmers generally experienced higher inhalation risks than non-competitive swimmers because of longer exposure durations and higher activity-related inhalation rates. The only exception or weaker contrast was observed for the C7–10 group under worst-case assumptions, where the upper-bound session duration assigned to non-competitive children was higher than that assigned to competitive children. This longer non-competitive exposure duration reduced the expected difference between swimmer groups, indicating that inhalation risk in young children is strongly influenced by the scenario-specific session-duration assumption.

When contrasted with earlier studies using the SWIMODEL toolbox (35), which produced higher risk estimates (10^–^^1^–10^–^^4^) due to strong dependence on Henry’s Law constant for air concentration modeling (1, 44, 54), our results are more consistent with studies applying alternative inhalation modeling approaches, which typically report risks on the order of 10^−5^ (47, 55–57).

Although SP-B is supplied by groundwater, typically associated with lower organic precursor levels, the TOC concentrations in SP-B nonetheless exceeded that of SP-A. Considering the facility characteristics and usage patterns previously described in the Materials section, the elevated TOC and DBP levels in SP-B may be associated with bather-related organic inputs. Although lower TOC might be expected from the groundwater source, the high proportion of child swimmers and the intensive bather load in SP-B may have contributed to increased organic matter inputs and higher DBP precursor availability. Consequently, the organic matter load by swimmers reversed the low-TOC signature of the groundwater source, leading to TOC levels in SP-B surpassing those in SP-A. This interpretation is further supported by the weekly swimmer-load, where SP-B exhibited a substantially higher bather density (1.19 n/m^3^.week) than SP-A (0.46 n/m^3^.week), suggesting that the elevated organic inputs driven by higher swimmer pressure likely contributed to the observed differences in DBP precursor load and associated exposure patterns.

### 3.5 Chronic toxic risk and aggregate cancer risk

The non-CR estimates calculated separately for the dermal, accidental ingestion, and buccal/sublingual pathways revealed clear contrasts between specified pools, swimmer activity profiles (competitive vs. non-competitive), and age groups. Unless otherwise specified, all numerical interpretations are based on the average levels of central-tendency estimates. Across the quantified water-contact routes, namely dermal contact, accidental ingestion, and buccal/sublingual exposure, competitive swimmers, particularly children, generally exhibited higher non-cancer risks due to increased exposure frequency and longer or more intensive session durations. Inhalation-related non-cancer risk was not quantified because suitable route-specific reference concentrations or inhalation reference doses were not available for the target DBPs; therefore, the non-cancer risk results should be interpreted as limited to the quantified water-contact pathways. In SP-A, dermal non-CRs remained below the threshold for all chemicals and swimmer groups. For instance, dermal CH non-CRs for competitive adults showed a central-tendency of 0.167 (upper-bound: 0.341), while children showed slightly higher values (C7–10 worst-case: 0.314, upper-bound: 0.642). Dermal CTRs of DCAA and TCAA in SP-A were even lower (adult competitive central-tendency values typically <10^−2^), indicating minimal chronic dermal toxicity under both typical and upper-bound exposure assumptions.

In SP-B, dermal non-CR profile diverged strongly by chemical class. Dermal CH risks were substantially higher than in SP-A (adult competitive central-tendency: 0.588; upper-bound: 1.10), and children exhibited even greater values (C7–10 central-tendency: 0.853; worst-case: 1.11; worst-case upper-bound: 2.07). However, dermal non-CRs for targeted HAAs in SP-B were extremely low across all swimmer categories, despite the markedly higher aqueous HAA concentrations in this pool. This seemingly paradoxical outcome is explained by the very low percutenous absorption rate (K_p_) of HAAs, which are approximately 1.90 × 10^−5^ m/h for both DCAA and TCAA, compared to 1.60 × 10^−3^ m/h for CH (58). Consequently, even though HAA concentrations in SP-B exceed those of CH, their transdermal transport potential is >80-fold lower, resulting in negligible absorbed dose and correspondingly minimal dermal non-CR. Thus, within the quantified dermal pathway, chronic toxicity in SP-B was driven almost exclusively by chloroform, whereas dermal contributions of DCAA and TCAA remained negligible under the evaluated dermal exposure scenarios. Non-competitive swimmers in both pools exhibited dermal non-CRs several orders of magnitude below the risk threshold.

The non-CR values estimated for the accidental ingestion pathway were consistently lower than dermal CH risks, regardless of pool or swimmer group. In SP-A, ingestion non-CRs for competitive adults were low (CH: 6.02 × 10^−4^; DCAA: 1.84 × 10^−3^; TCAA: 3.95 × 10^−4^), relatively higher values appeared in children (C7–10 worst-case ingestion non-CRs up to 3.60 × 10^−2^ for DCAA). In SP-B, ingestion non-CRs increased but remained well below the threshold. Competitive children aged 7–10 had the highest ingestion non-CR values (e.g., DCAA: 2.66 × 10^−2^ central-tendency; 8.45 × 10^−2^ worst-case; 95th percentile: 1.53 × 10^−1^), while adult ingestion non-CRs rarely exceeded 2 × 10^−2^ even under worst-case conditions. Non-competitive swimmers in both pools showed ingestion non-CRs within the 10^−6^ –10^−3^ range.

The buccal/sublingual exposure pathway displayed patterns similar to ingestion, but with slightly lower magnitudes. In SP-A, competitive adult buccal/sublingual non-CRs ranged from 4.70 × 10^−4^ to 2.19 × 10^−3^, with children again showing higher values (C7–10 worst-case up to 1.50 × 10^−2^ for DCAA). SP-B produced larger buccal/sublingual non-CRs for competitive swimmers, especially children (e.g., TCAA C7–10 worst-case: 4.07 × 10^−2^; 95th percentile: 6.82 × 10^−2^), while adults remained below 10^−2^. All non-competitive groups exhibited buccal/sublingual non-CRRs in the range of 10^−6^–10^−3^.

Collectively, the three-route analysis showed that dermal CH exposure overwhelmingly dominated chronic toxic risk in SP-B, whereas DCAA and TCAA contributed only marginally due to their extremely low dermal permeabilities despite higher water concentrations. In SP-A, where dermal risks were lower, oral routes (ingestion + buccal) contributed a comparatively larger share to the aggregate non-CR of each stressor in children; however, the cumulative non-CR across all DBP stressors remained far below the risk threshold. Across the quantified water-contact scenarios, non-competitive swimmers exhibited low non-cancer risk values through dermal, accidental ingestion, and buccal/sublingual pathways. These results indicate that pool DBP levels, swimming intensity, and age-related physiological factors influence the magnitude and distribution of quantified chronic toxic risk; however, inhalation-related non-cancer risk remains unquantified.

Aggregate carcinogenic risks calculated by summing the quantified exposure routes for each individual DBP are presented separately for SP-A and SP-B in Tables 1, 2, respectively. Route-share analysis revealed distinct chemical-specific exposure patterns shaped by the physicochemical properties of each DBP, swimmer activity, and age-dependent physiological factors (Supplementary Table S5). For CF, all quantified routes included ingestion, dermal contact, buccal/sublingual exposure, and inhalation; therefore, the route-share results indicate an inhalation-dominated profile, with inhalation contributing approximately 89–96% of aggregate CF carcinogenic risk and dermal uptake accounting for approximately 4–11%, while oral routes contributed less than 0.5%. This pattern reflects CH’s high volatility and relatively high dermal permeability, both of which magnify airborne and dermal transfer compared with oral pathways. In contrast, DCAA and TCAA displayed different route-share structures among the quantified routes only, because inhalation risks for these HAAs could not be estimated. Within this quantified framework, oral routes, including accidental ingestion and buccal/sublingual exposure, contributed approximately 40–75% of the calculated HAA carcinogenic risk, whereas dermal uptake accounted for approximately 8–42%. Therefore, the route-share results for DCAA and TCAA should not be interpreted as complete total-risk profiles, but rather as distributions among the quantified ingestion, dermal, and buccal/sublingual pathways. This pattern is partly attributable to the lower dermal permeability coefficients of HAAs relative to CF, which limit transdermal transport. However, the route-share results for DCAA and TCAA should be interpreted only among the quantified carcinogenic-risk pathways. Because DCAA and TCAA exhibit very low volatility, their pool-air concentrations were not modeled; therefore, possible aerosol-mediated inhalation exposure during splashing may not be fully captured. In addition, inhalation cancer risks for DCAA and TCAA could not be quantified because inhalation unit-risk values or inhalation slope factors were not available for these compounds. Literature evidence from swimming pool exposure assessments indicates that ingestion is the dominant exposure pathway for haloacetic acids, accounting for approximately 94% of total exposure, while inhalation contributes a smaller but non-negligible fraction (approximately 5%), depending on pool conditions and activity patterns (59). Consequently, the HAA route-share outcomes represent only the relative contributions of accidental ingestion, buccal/sublingual exposure, and dermal contact, and should not be interpreted as complete total-risk profiles including pool-air exposure. These findings highlight the need for future toxicological studies to develop inhalation toxicity parameters, including inhalation slope factors or unit-risk values, for haloacetic acids to enable more comprehensive risk characterization.

**Table 1:** Aggregate carcinogenic risks in SP-A.

Swimmer group	Age group	SP-A		
		CF	DCAA	TCAA
Competitive	Adult (CT)	2.31 × 10^−4^	5.73 × 10^−7^	8.62 × 10^−7^
	Adult (WC)	3.00 × 10^−4^	1.07 × 10^−6^	1.60 × 10^−6^
	C 11–14 (CT)	6.46 × 10^−5^	2.15 × 10^−7^	3.16 × 10^−7^
	C 11–14 (WC)	8.38 × 10^−5^	4.08 × 10^−7^	6.04 × 10^−7^
	C 7–10 (CT)	8.68 × 10^−5^	3.28 × 10^−7^	4.93 × 10^−7^
	C 7–10 (WC)	1.13 × 10^−4^	6.24 × 10^−7^	9.71 × 10^−7^
Non-competitive	Adult (CT)	2.08 × 10^−6^	1.27 × 10^−8^	1.90 × 10^−8^
	Adult (WC)	8.37 × 10^−6^	8.25 × 10^−8^	1.24 × 10^−7^
	C 11–14 (CT)	1.92 × 10^−6^	1.27 × 10^−8^	1.91 × 10^−8^
	C 11–14 (WC)	2.50 × 10^−6^	2.58 × 10^−8^	3.88 × 10^−8^
	C 7–10 (CT)	2.86 × 10^−6^	2.13 × 10^−8^	3.30 × 10^−8^
	C 7–10 (WC)	3.43 × 10^−6^	4.14 × 10^−8^	6.34 × 10^−8^

The ingestion route represents the accidental ingestion of pool water, whereas the buccal/sublingual route reflects non-ingestive oral exposure occurring through contact of water with the oral mucosa. Values represent route-summed carcinogenic risks for each individual DBP and should be interpreted as conservative upper-bound screening estimates. Because DBP classes may differ in toxicological modes of action and mixture-interaction data are unavailable, cumulative risks across different DBPs should not be interpreted as absolute total mixture-risk values.

**Table 2:** Aggregate carcinogenic risks in SP-B.

Swimmer group	Age group	SP-B		
		CF	DCAA	TCAA
Competitive	Adult (CT)	8.15 × 10^−4^	1.35 × 10^−6^	1.09 × 10^−5^
	Adult (WC)	1.06 × 10^−3^	2.50 × 10^−6^	2.03 × 10^−5^
	C 11–14 (CT)	2.27 × 10^−4^	5.05 × 10^−7^	4.09 × 10^−6^
	C 11–14 (WC)	2.95 × 10^−4^	9.59 × 10^−7^	7.77 × 10^−6^
	C 7–10 (CT)	3.06 × 10^−4^	7.70 × 10^−7^	6.24 × 10^−6^
	C 7–10 (WC)	3.97 × 10^−4^	1.52 × 10^−6^	1.23 × 10^−5^
Non-competitive	Adult (CT)	7.31 × 10^−6^	2.97 × 10^−8^	2.41 × 10^−7^
	Adult (WC)	2.95 × 10^−5^	1.94 × 10^−7^	1.57 × 10^−6^
	C 11–14 (CT)	6.75 × 10^−6^	2.98 × 10^−8^	2.41 × 10^−7^
	C 11–14 (WC)	8.81 × 10^−6^	6.06 × 10^−8^	4.91 × 10^−7^
	C 7–10 (CT)	1.01 × 10^−5^	5.15 × 10^−8^	4.17 × 10^−7^
	C 7–10 (WC)	1.21 × 10^−5^	9.89 × 10^−8^	8.01 × 10^−7^

The ingestion route represents the accidental ingestion of pool water, whereas the buccal/sublingual route reflects non-ingestive oral exposure occurring through contact of water with the oral mucosa. Values represent route-summed carcinogenic risks for each individual DBP and should be interpreted as conservative upper-bound screening estimates. Because DBP classes may differ in toxicological modes of action and mixture-interaction data are unavailable, cumulative risks across different DBPs should not be interpreted as absolute total mixture-risk values.

Consistent with previous research, chloroform exhibited risk levels comparable to earlier reports in the literature (54, 60). In our assessment, although dermal and oral routes contributed measurably to chloroform exposure, the inhalation route overwhelmingly dominated aggregate non-CR, typically accounting for ≈89–96% of total exposure across all swimmer groups. This inhalation dominance aligns closely with earlier findings that reported chloroform carcinogenic risks ranging from 5.10 × 10^−5^ to 1.00 × 10^−6^ for ingestion and dermal routes, but substantially higher values of 1.00 × 10^−4^ to 5.10 × 10^−5^ for inhalation (61). The close agreement between our inhalation-driven route contributions and these literature-reported cancer risk ranges confirms the robustness of the multi-route exposure structure observed in swimming-pool environments. Overall, our results reinforce the well-established pattern that chloroform risk is primarily inhalation-driven, with dermal exposure serving as a secondary contributor and oral routes exerting only minor influence, consistent with the previous body of evidence.

Importantly, the buccal/sublingual route, often ignored or collapsed into ingestion in many exposure assessments, was found to produce health-risk levels comparable to accidental ingestion for targeted priority DBPs, particularly among children and competitive swimmers who exhibit higher exposure frequency and longer activity durations. This finding highlights that non-ingestive oral uptake through contact of pool water with the oral mucosa represents a non-trivial exposure route, capable of contributing 20–45% of total HAA uptake in several scenarios. Such evidence indicates that buccal/sublingual exposure should not be dismissed as negligible: although typically unmeasured and unregulated, it can meaningfully influence aggregate non-CR, especially for chemicals with low dermal permeability but appreciable aqueous concentrations. Collectively, these patterns underscore that chloroform risk is driven almost entirely by inhalation (and secondarily dermal), whereas HAA risks are predominantly oral, with dermal contributions inherently limited by permeability and inhalation contributions unquantified due to toxicological data gaps. These results emphasize that DBP-specific physicochemical properties, rather than bulk concentration alone, govern the relative importance of inhalation, dermal, and oral exposure pathways in swimming-pool environments.

## 4 Conclusion

This study shows that carcinogenic risks from DBPs in swimming pools are strongly route- and chemical-specific. Chloroform risks were dominated by inhalation, exceeding the *de minimis* level across swimmer groups and aligning with previous evidence. In contrast, DCAA and TCAA risks arose mainly from oral exposure, as their low volatility and very low dermal permeability limited inhalation and dermal contributions. SP-B generally showed higher cancer risk estimates than SP-A for the selected DBPs, with TCAA being the main HAA-related contributor, particularly among competitive swimmers. The buccal/sublingual route, often ignored in swimmer risk assessments, produced risk levels comparable to accidental ingestion, particularly among children and competitive swimmers. Competitive swimmers experienced the highest risks overall due to longer exposure durations and higher inhalation rates. For non-cancer risk, hazard quotients calculated for the quantified water-contact routes were generally below the selected concern threshold, but dermal chloroform in SP-B exceeded HQ = 1 under some upper-bound and worst-case assumptions. Inhalation-related non-cancer risk could not be quantified because suitable route-specific toxicity values were unavailable. Therefore, the chronic toxicity findings should be interpreted as route-specific estimates rather than as a complete assessment of total non-cancer risk across all water and pool-air exposure pathways.

Although the filling water TOC concentrations were lower in SP-B, the higher DBP levels measured in this pool resulted in elevated cancer risks, indicating that source water quality alone does not dictate DBP-related health outcomes. This discrepancy highlights that swimmer profile and bather load can exert greater influence on DBP formation than the precursor contribution from filling water. Consequently, risk-reduction strategies must prioritize bather-related factors, particularly hygiene practices such as pre-swim showering, removal of personal care products, and minimizing organic nitrogen inputs, alongside chemical and engineering controls. These findings highlight the need for DBP-specific, route-resolved risk assessments and emphasize that targeted DBP control, especially reducing TCAA precursors and improving ventilation for CF, will be essential for minimizing swimmer exposure in indoor pool environments.

## Data Availability

The original contributions presented in the study are included in the article/Supplementary material, further inquiries can be directed to the corresponding author.
