# Supplementary material for: Route-resolved priority risks of disinfection by-products in indoor pools: the underestimated role of buccal/sublingual exposure for the public and competitive swimmers
Source: Front Public Health. 2026 Jul 28;14:1896577. doi: 10.3389/fpubh.2026.1896577 (PMC13457292; doi:10.3389/fpubh.2026.1896577)
Supplement: Supplementary file 1 [file Table_1.docx]

**Supporting Information**

**for**

**Route‑resolved priority risks of disinfection by‑products in indoor pools: the underestimated role of buccal/sublingual exposure for the public and competitive swimmers**

Mesut Genişoğlu^1^, Mert Minaz^2*^, Bilgehan İlker Harman^3^, Sait C. Sofuoglu^1^

^1^ Department of Environmental Engineering, Izmir Institute of Technology, Izmir, Türkiye

^2^ Department of Aquaculture, Faculty of Fisheries, Recep Tayyip Erdoğan University, Rize, Türkiye

^3^ Department of Environmental Engineering, Suleyman Demirel University, Isparta, Türkiye

*Corresponding author: [mert.minaz@erdogan.edu.tr](mailto:mert.minaz@erdogan.edu.tr)

**Table SI1.** DBP concentration for both pools (μg/L) in water and estimated indoor air (μg/m^3^) concentrations.

| SP A | | | | |
| --- | --- | --- | --- | --- |
|  | DBP concentrations in pool water (28) | | | Estimated indoor air concentration |
|  | CF | DCAA | TCAA | CF |
| N | 48 | 48 | 48 |  |
| Mean | 19.6 | 23.9 | 25.7 | 82.9 |
| SD | 9.51 | 8.60 | 16.8 | 40.2 |
| 5th | 8.74 | 15.3 | 9.68 | 36.9 |
| 25th | 12.3 | 19.1 | 13.6 | 52.2 |
| Median | 16.8 | 22.4 | 20.2 | 71.2 |
| 75th | 25.1 | 25.8 | 31.6 | 106 |
| 95th | 39.3 | 40.3 | 60.4 | 166 |
| SP B | | | | |
| N | 48 | 48 | 48 |  |
| Mean | 68.9 | 56.1 | 325 | 292 |
| SD | 29.1 | 21.9 | 168 | 123 |
| 5th | 22.5 | 30.8 | 31.1 | 95.1 |
| 25th | 50.6 | 41.4 | 197 | 214 |
| Median | 67.7 | 56.8 | 385 | 286 |
| 75th | 86.7 | 67.6 | 440 | 367 |
| 95th | 110 | 93.5 | 536 | 466 |

**Table SI2.** Values of the constants used in risk estimation for the three DBPs.

|  | | CF | DCAA | TCAA |
| --- | --- | --- | --- | --- |
| SF [(mg/kg/d)^-1^] | Inhalation | ^a^8.05×10^-2^ | - | - |
|  | Dermal Contact | ^a^3.05×10^-2^ | ^b^5.00×10^-2^  ^b^5.00×10^-2^ | ^b^7.00×10^-2^  ^b^7.00×10^-2^ |
|  | Ingestion | ^a^6.10×10^-3^ |  |  |
| RfD (mg/kg/d) | Ingestion | ^c^1.00×10^-2^  ^c^1.00×10^-2^ | ^d^4.00×10^-3^  ^d^4.00×10^-3^ | ^e^2.00×10^-2^  ^e^2.00×10^-2^ |
|  | Dermal Contact |  |  |  |
| Kp (cm/h) | | ^f^1.60×10^-1^ | ^f^1.90×10^-3^ | ^f^1.90×10^-3^ |
| a,b(34), c,d,e(33, 37, 38), f(58) | | | | |
| a: Derived from inhalation unit risk.  b: Values obtained from RAIS (34).  c–e: Values obtained from USEPA IRIS (33, 37, 38).  f: Permeability coefficients obtained from (58).  CF: chloroform; DCAA: dichloroacetic acid; TCAA: trichloroacetic acid; SF: slope factor; RfD: reference dose; Kp: dermal permeability coefficient. | | | | |

**Table SI3.** Scenario-specific input variables used in the exposure and oral-route risk calculations..

| **Swimmer group** | **Age group** | **Scenario** | **ED (yr)** | **Session duration (h/week)** | **EF (week/year)** | **IngR (L/h)** | **WI for buccal/sublingual route (L/h)** | **ARBuccal/Sublingual** | **BW (kg)** | **LT (yr)** |
| --- | --- | --- | --- | --- | --- | --- | --- | --- | --- | --- |
| Competitive | Adult | CT | 22 | 16.2 | 48 | 0.0105 | 1.25 | 0.01 | 72.8 | 77.3 |
| Competitive | Adult | WC/Upper-bound | 22 | 21.0 | 48 | 0.0355 | 1.25 | 0.01 | 72.8 | 77.3 |
| Competitive | C11–14 | CT | 4 | 16.2 | 48 | 0.0245 | 2.50 | 0.01 | 46.8 | 77.3 |
| Competitive | C11–14 | WC/Upper-bound | 4 | 21.0 | 48 | 0.0600 | 2.50 | 0.01 | 46.8 | 77.3 |
| Competitive | C7–10 | CT | 4 | 16.2 | 48 | 0.0245 | 2.50 | 0.01 | 27.5 | 77.3 |
| Competitive | C7–10 | WC/Upper-bound | 4 | 21.0 | 48 | 0.0600 | 2.50 | 0.01 | 27.5 | 77.3 |
| Non-competitive | Adult | CT | 30 | 1.0 | 36.2 | 0.0210 | 2.50 | 0.01 | 72.8 | 77.3 |
| Non-competitive | Adult | WC/Upper-bound | 30 | 9.0 | 36.2 | 0.0710 | 2.50 | 0.01 | 72.8 | 77.3 |
| Non-competitive | C11–14 | CT | 4 | 1.0 | 36.2 | 0.0490 | 5.00 | 0.01 | 46.8 | 77.3 |
| Non-competitive | C11–14 | WC/Upper-bound | 4 | 27.8 | 36.2 | 0.1200 | 5.00 | 0.01 | 46.8 | 77.3 |
| Non-competitive | C7–10 | CT | 4 | 1.0 | 36.2 | 0.0490 | 5.00 | 0.01 | 27.5 | 77.3 |
| Non-competitive | C7–10 | WC/Upper-bound | 4 | 30.2 | 36.2 | 0.1200 | 5.00 | 0.01 | 27.5 | 77.3 |

**Note:** CT: central tendency; WC: worst-case; ED: exposure duration; EF: exposure frequency; IngR: accidental ingestion rate; WI: water intake parameter for buccal/sublingual exposure; ARBuccal/Sublingual: absorption factor for buccal/sublingual exposure; BW: body weight; LT: lifetime. CT scenarios used mean session duration and mean ingestion-rate values, whereas WC scenarios used upper-bound session duration and upper-bound ingestion-rate values when available. Buccal/sublingual exposure was calculated using WI × ARBuccal/Sublingual.

***Body Surface Area Calculation Method***

Body surface areas of different swimmers were calculated using Du Bois empirical model (40)

$SA=\left( W^{0.425}\times H^{0.725} \right)\times0.007184$	Eq. SI1

*where, SA: skin surface area (m^2^); W: weight (kg); H: height (cm).*

**Table SI4.** Detection frequencies and selection status of THMs and HAAs considered in the quantitative risk assessment.

| **DBP group** | **Compound** | **Abbreviation** | **Detection frequency in SP-A (%)** | **Detection frequency in SP-B (%)** | **Required risk parameters** | **Included in quantitative risk assessment?** | **Reason** |
| --- | --- | --- | --- | --- | --- | --- | --- |
| THM | Chloroform | CHCl₃ / CF | 100 | 100 | Available | Yes | Detection frequency >50% in both pools and required parameters were available |
| THM | Bromodichloromethane | CHCl₂Br | 0 | 0 | Not evaluated | No | Detection frequency criterion was not met |
| THM | Dibromochloromethane | CHClBr₂ | 0 | 0 | Not evaluated | No | Detection frequency criterion was not met |
| HAA | Chloroacetic acid | CAA | 31.25 | 12.5 | Not evaluated | No | Detection frequency criterion was not met |
| HAA | Dichloroacetic acid | DCAA | 100 | 100 | Available | Yes | Detection frequency >50% in both pools and required parameters were available |
| HAA | Bromochloroacetic acid | BCAA | 25 | 29.17 | Not evaluated | No | Detection frequency criterion was not met |
| HAA | Trichloroacetic acid | TCAA | 100 | 100 | Available | Yes | Detection frequency >50% and required oral/dermal parameters were available |
| HAA | Dibromochloroacetic acid | DBCAA | 0 | 4.16 | Not evaluated | No | Detection frequency criterion was not met |
| HAA | Bromodichloroacetic acid | BDCAA | 6.25 | 6.25 | Not evaluated | No | Detection frequency criterion was not met |

**Note:** “Available” indicates that the toxicity/exposure parameters required for the quantitative route-resolved risk assessment were available for the routes evaluated in this study. “Not evaluated” indicates that the compound was not further assessed for parameter availability because it did not meet the detection-frequency criterion.

**Table SI5.** Route shares for aggregate carcinogenic risks in SP-A and SP-B.

|  | | | Route Share for SP-A (%) | | | | Route Share for SP-B (%) | | | |
| --- | --- | --- | --- | --- | --- | --- | --- | --- | --- | --- |
|  |  |  | Ingestion* | Dermal | Buccal / Sublingual* | Inhalation* | Ingestion | Dermal | Buccal / Sublingual* | Inhalation |
| **CF** | Adult (CT) | Competitive | 0.02 | 6.37 | 0.03 | 93.6 | 0.02 | 6.36 | 0.03 | 93.6 |
|  | Adult (WC) |  | 0.08 | 6.36 | 0.03 | 93.5 | 0.08 | 6.36 | 0.03 | 93.5 |
|  | C 11-14 (CT) |  | 0.05 | 5.04 | 0.06 | 94.9 | 0.05 | 5.03 | 0.06 | 94.9 |
|  | C 11-14 (WC) |  | 0.13 | 5.03 | 0.06 | 94.8 | 0.13 | 5.03 | 0.06 | 94.8 |
|  | C 7-10 (CT) |  | 0.07 | 4.48 | 0.07 | 95.4 | 0.07 | 4.48 | 0.07 | 95.4 |
|  | C 7-10 (WC) |  | 0.17 | 4.47 | 0.07 | 95.3 | 0.17 | 4.47 | 0.07 | 95.3 |
|  | Adult (CT) | Non-Competitive | 0.08 | 11.2 | 0.10 | 88.6 | 0.08 | 11.19 | 0.10 | 88.6 |
|  | Adult (WC) |  | 0.27 | 11.2 | 0.10 | 88.5 | 0.27 | 11.17 | 0.10 | 88.5 |
|  | C 11-14 (CT) |  | 0.13 | 6.06 | 0.13 | 93.7 | 0.13 | 6.06 | 0.13 | 93.7 |
|  | C 11-14 (WC) |  | 0.32 | 6.05 | 0.13 | 93.5 | 0.32 | 6.05 | 0.13 | 93.5 |
|  | C 7-10 (CT) |  | 0.16 | 5.29 | 0.17 | 94.4 | 0.16 | 5.28 | 0.17 | 94.4 |
|  | C 7-10 (WC) |  | 0.40 | 5.27 | 0.16 | 94.2 | 0.40 | 5.27 | 0.16 | 94.2 |
| **DCAA** | Adult (CT) | Competitive | 18.2 | 60.1 | 21.7 | NA | 18.2 | 60.1 | 21.7 | NA |
|  | Adult (WC) |  | 43.0 | 41.9 | 15.1 | NA | 43.0 | 41.9 | 15.1 | NA |
|  | C 11-14 (CT) |  | 32.0 | 35.3 | 32.7 | NA | 32.0 | 35.3 | 32.7 | NA |
|  | C 11-14 (WC) |  | 53.6 | 24.1 | 22.3 | NA | 53.6 | 24.1 | 22.3 | NA |
|  | C 7-10 (CT) |  | 35.8 | 27.7 | 36.5 | NA | 35.8 | 27.7 | 36.5 | NA |
|  | C 7-10 (WC) |  | 59.7 | 15.5 | 24.9 | NA | 55.4 | 21.5 | 23.1 | NA |
|  | Adult (CT) | Non-Competitive | 26.1 | 42.9 | 31.0 | NA | 26.1 | 42.9 | 31.0 | NA |
|  | Adult (WC) |  | 54.4 | 26.5 | 19.1 | NA | 54.4 | 26.5 | 19.1 | NA |
|  | C 11-14 (CT) |  | 38.9 | 21.4 | 39.7 | NA | 38.9 | 21.4 | 39.7 | NA |
|  | C 11-14 (WC) |  | 60.9 | 13.7 | 25.4 | NA | 60.9 | 13.7 | 25.4 | NA |
|  | C 7-10 (CT) |  | 42.8 | 13.6 | 43.6 | NA | 40.1 | 19.1 | 40.9 | NA |
|  | C 7-10 (WC) |  | 64.7 | 8.4 | 26.9 | NA | 62.1 | 12.1 | 25.9 | NA |
| **TCAA** | Adult (CT) | Competitive | 18.2 | 60.1 | 21.7 | NA | 18.2 | 60.1 | 21.7 | NA |
|  | Adult (WC) |  | 43.0 | 41.9 | 15.1 | NA | 43.0 | 41.9 | 15.1 | NA |
|  | C 11-14 (CT) |  | 32.8 | 33.7 | 33.5 | NA | 45.2 | 8.6 | 46.1 | NA |
|  | C 11-14 (WC) |  | 54.5 | 22.8 | 22.7 | NA | 66.9 | 5.2 | 27.9 | NA |
|  | C 7-10 (CT) |  | 35.8 | 27.7 | 36.5 | NA | 35.8 | 27.7 | 36.5 | NA |
|  | C 7-10 (WC) |  | 57.7 | 18.3 | 24.0 | NA | 57.7 | 18.3 | 24.0 | NA |
|  | Adult (CT) | Non-Competitive | 26.1 | 42.9 | 31.0 | NA | 26.1 | 42.9 | 31.0 | NA |
|  | Adult (WC) |  | 54.4 | 26.5 | 19.1 | NA | 54.4 | 26.5 | 19.1 | NA |
|  | C 11-14 (CT) |  | 38.9 | 21.4 | 39.7 | NA | 38.9 | 21.4 | 39.7 | NA |
|  | C 11-14 (WC) |  | 60.9 | 13.7 | 25.4 | NA | 60.9 | 13.7 | 25.4 | NA |
|  | C 7-10 (CT) |  | 41.5 | 16.1 | 42.4 | NA | 41.5 | 16.1 | 42.4 | NA |
|  | C 7-10 (WC) |  | 63.5 | 10.0 | 26.4 | NA | 63.5 | 10.0 | 26.5 | NA |
| **The ingestion route represents the accidental ingestion of pool water, whereas the buccal/sublingual route reflects non‑ingestive oral exposure occurring through contact of water with the oral mucosa.* | | | | | | | | | | |

**References**

28.	Genisoglu M, Minaz M, Tanacan E, Sofuoglu SC, Kaplan-Bekaroglu SS, Kanan A, Ates N, Sardohan-Koseoglu T, Yigit NÖ, Harman BI. Halogenated By-Products in Chlorinated Indoor Swimming Pools: A Long-Term Monitoring and Empirical Modeling Study. *ACS Omega* (2023) 8:11364–11372. doi: 10.1021/ACSOMEGA.3C00091/ASSET/IMAGES/LARGE/AO3C00091_0006.JPEG

34.	RAIS. The Risk Assessment Information System. (2022) https://rais.ornl.gov/cgi-bin/tools/TOX_search?select=chemspef [Accessed May 1, 2022]

33.	USEPA. Integrated Risk Information System. (2026) https://www.epa.gov/iris [Accessed May 31, 2026]

35.	USEPA. Swimmer Exposure Assessment Model (SWIMODEL) Version 3.0: User’s Manual. (2003).

58.	Xu X, Mariano TM, Laskin JD, Weisel CP. Percutaneous Absorption of Trihalomethanes, Haloacetic Acids, and Haloketones. *Toxicol Appl Pharmacol* (2002) 184:19–26. doi: 10.1006/TAAP.2002.9494

40.	Du Bois D, Du Bois EF. A height-weight formula to estimate the surface area of man. *Proc Soc Exp Biol Med* (1916) 13:77–78. doi: 10.3181/00379727-13-45
